# Enrichment of Exosome-Like Extracellular Vesicles from Plasma Suitable for Clinical Vesicular miRNA Biomarker Research

**DOI:** 10.3390/jcm8111995

**Published:** 2019-11-15

**Authors:** Sohee Moon, Dong Wun Shin, Sujin Kim, Young-Sun Lee, Sakulrat Mankhong, Seong Wook Yang, Phil Hyu Lee, Dong-Ho Park, Hyo-Bum Kwak, Jae-Sun Lee, Ju-Hee Kang

**Affiliations:** 1 Department of Pharmacology, College of Medicine, Inha University, Incheon 22212, Korea; 2 Hypoxia-Related Disease Research Center, College of Medicine, Inha University, Incheon 22212, Korea; 3 Department of Emergency Medicine, Inje University Ilsan Paik Hospital, Goyang 10380, Korea; 4 Department of Kinesiology, Inha University, Incheon 22212, Korea; 5 Department of Systems Biology, College of Life Science and Biotechnology, Yonsei University, Seoul 03722, Korea; 6 Department of Neurology, Yonsei University College of Medicine, Seoul 03722, Korea; 7 Department of Molecular Medicine, College of Medicine, Inha University, Incheon 22212, Korea

**Keywords:** extracellular vesicle, plasma miRNA, biomarker, polymer-precipitation, proteinase K

## Abstract

Exosome-like extracellular vesicles (ELVs) contain biomolecules that have potential as diagnostic biomarkers, such as proteins, micro-RNAs (miRNAs), and lipids. However, it is difficult to enrich ELVs consistently with high yield and purity from clinical samples, which hampers the development of ELV biomarkers. This is particularly true for miRNAs in protein-rich plasma. Hence, we modified ELV isolation protocols of three commercially available polymer-precipitation-based kits using proteinase K (PK) treatment to quantify ELV-associated miRNAs in human plasma. We compared the yield, purity, and characteristics of enriched plasma ELVs, and measured the relative quantity of three selected miRNAs (miR-30c, miR-126, and miR-192) in ELVs using six human plasma samples. Compared with the original protocols, we demonstrated that ELVs can be isolated with PK treatment with high purity (i.e., lack of non-exosomal proteins and homogeneous size of vesicles) and yield (i.e., abundancy of exosomal markers), which were dependent on kits. Using the kit with the highest purity and yield with PK treatment, we successfully quantified ELV miRNAs (levels of 45%–65% in total plasma) with acceptable variability. Collectively, ELV enrichment using the modified easy-to-use method appears suitable for the analysis of miRNAs, although its clinical applicability needs to be confirmed in larger clinical studies.

## 1. Introduction

Exosomes are extracellular vesicles (EVs) with a diameter of approximately 30–150 nm and a density of 1.13~1.19 g/mL that are secreted from most cells through sorting pathways. Interest in the biologic functions of exosomes and the molecular contents of exosomes, including nucleic acids, proteins, and lipids, is growing exponentially. EVs are found in biologic fluids such as blood, plasma, urine, saliva, milk, and cerebrospinal fluid, and their biologic contents can be used as diagnostic biomarkers or therapeutic targets [1]. Although various methods, including differential ultracentrifugation (UC), density-gradient ultracentrifugation (DG-UC), size-exclusion chromatography (SEC), and polymer-based precipitation (PP), allow exosome-like extracellular vesicles (ELVs) to be isolated from biologic fluids or cell culture media, several issues remain unresolved, especially in terms of clinical applications, including the aggregation of vesicles, low recovery, necessity of a large sample volume, and contamination with soluble proteins and lipoproteins [2,3]. In particular, co-isolation with ELV-sized lipoproteins or protein precipitates can interfere with the analysis of ELV-associated micro-RNAs (miRNAs), because certain lipoproteins (e.g., high-density lipoprotein (HDL)) may carry miRNAs [4].

miRNAs are small non-coding RNAs, approximately 22 nucleotides long, that regulate various biologic processes by binding to the complementary sites of the 3′-untranslated regions of target messenger RNAs (mRNAs) and silencing them translationally or by mRNA degradation [5]. Circulating miRNAs are protected from ribonucleases by binding to RNA-binding proteins [6,7,8] or by selective encapsulation by ELVs [9], which increases the stability of miRNAs in biofluids. Considering the sorting of miRNAs into ELVs, several mechanisms of selective miRNA loading into exosomes have been postulated [10,11], and it has been increasingly recognized that the quantity of specific miRNAs or miRNA profiles in ELVs are altered in certain disease states [12,13,14,15,16,17]. Therefore, ELV-associated miRNAs might be potential biomarkers for disease diagnosis; they could also be used to monitor disease progression [18,19,20] or as therapeutic targets [21].

Various methods to isolate ELVs and to profile ELV-associated miRNAs in body fluids for disease diagnosis are available, including UC, DG-UC, SEC, and PP [22]. The method most commonly used is ELV isolation by differential UC, which is relatively simple and cheap [23]. However, this technique is time-consuming and results in a low recovery rate. It requires an ultracentrifuge, and ELVs may get mechanically damaged. Additionally, misinterpretation of biomarker levels may occur because of co-sediments such as non-specific particles (e.g., protein aggregates) or the non-specific binding of molecules to the surface of exosomes [24,25]. If necessary, DG-UC can be applied, which yields the purest fraction of exosomes with fewer contaminations when compared with UC. However, DG-UC is time-consuming and difficult to use. The ELV yield is low, and there is an apparent lack of reproducibility between laboratories, which limits its clinical use [26]. SEC separates intact vesicles depending on particle size using an SEC column. This method is relatively simple and can purify exosomes of regular shape. However, it is possible that other vesicles with similar sizes or lipoproteins are co-isolated [27], and the exosome yield is low [22].

PP is one of the most commonly used exosome enrichment methods. Commercially available kits are easy, fast, and simple; provide a high-throughput method for small sample volumes; and are the most reliable method used for clinical applications. However, previous studies have argued that PP provides low purity because of the co-precipitation of contaminants such as protein aggregates [22,26]. Therefore, the results obtained from exosomes co-isolated via a commercialized PP kit might be questionable. In particular, miRNA can be transported in plasma in non-vesicular form by binding to HDL [7,28] and argonaute proteins [6,29]. Non-vesicular and vesicular miRNAs need to be discriminated to develop ELV miRNA biomarkers.

In the present study, we modified the ELV isolation methods provided by the manufacturers of PP kits by adding protease K (PK) and acidification to maximize the advantages of PP-based ELV isolation and minimize contamination with non-vesicular miRNA. We evaluated ELV purity by detecting representative exosomal markers and non-exosomal proteins, morphological assessment by transmission electron microscopy (TEM), and the size distribution of the vesicles. In addition, we tested the effects of acidification of the samples on ELV protein quantities and the expression of exosomal markers. Furthermore, we quantified specific miRNAs using quantitative polymerase chain reaction (qPCR) in ELVs isolated from human plasma.

## 2. Materials and Methods

### 2.1. Blood Collection and Plasma Preparation

Because anticoagulants are a pre-analytical variable for the ELV yield [30] or subsequent qPCR [31], we selected ethylenediaminetetraacetic acid (EDTA), the most commonly used anticoagulant, to prepare the plasma. Fasting blood was drawn from the antecubital vein into EDTA tubes (BD Vacutainer K2E-EDTA; Beckton and Dickinson, Franklin Lake, NJ) from young healthy volunteers and immediately centrifuged at 3000× *g* for 10 min at room temperature (RT). The plasma was transferred to a new polypropylene tube and centrifuged again at 10,000× *g* for 20 min at RT to remove platelets, cell debris, and large particles. The supernatant was transferred to a new tube without disturbing the pellets, aliquoted, and then stored at −70 °C until use. For miRNA analysis in ELVs isolated from plasma, six elderly subjects (three cognitively normal elderly subjects and three patients with mild Alzheimer’s disease (AD)) agreed to participate in the study and donated blood. The demographic characteristics and clinical informations of the subjects are summarized in Supplementary Table S1. The Institutional Review Board and Ethical Committee of Inha University Hospital approved the study (approval No.: INHAUH2016-06-010-002). Written informed consent to participate was obtained from all volunteers.

### 2.2. ELV Isolation

**Figure 1:**
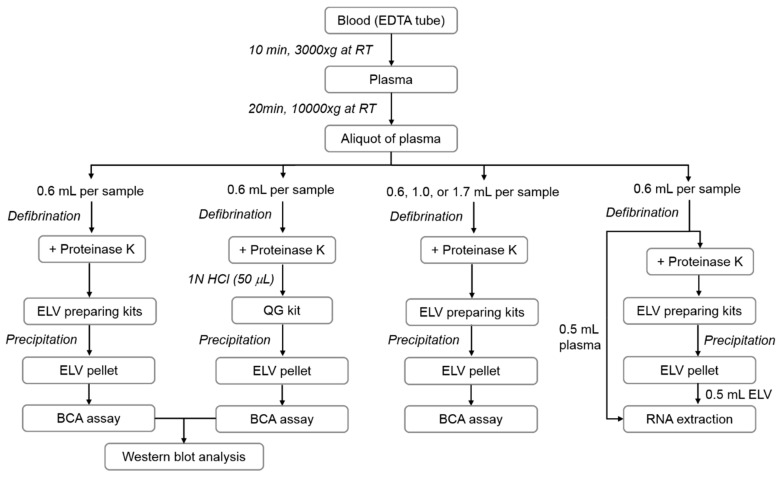
Schematic overview of experimental procedures with different protocols to isolate plasma extracellular vesicles (ELVs). EDTA, ethylenediaminetetraacetic acid; RT, room termperature; QG kit, miRCURY Exosome Serum/Plasma Kit.

We used three commercially available PP-based kits to isolate ELVs: the ExoQuick Exosome Isolation Kit for Serum and Plasma (SBI, #EXOQ5A-1; System Biosciences, Palo Alto, CA), the Invitrogen™ Total Exosome Isolation Kit (LT, #4484450; Thermo Fisher Scientific, Waltham, MA), and the miRCURY Exosome Serum/Plasma Kit (QG, #76603; Qiagen, Venlo, Netherlands). The basic procedures of ELV isolation were carried out according to the manufacturers’ protocols. Briefly, plasma was incubated with thromboplastin-D (for SBI kit, #100356; Thermo Fisher Scientific) or thrombin (for QG kit, #36402.01; SERVA, Heidelberg, Germany) for defibrination, followed by a precipitation procedure. For the LT kit, plasma was diluted with phosphate-buffered saline (PBS) following the manufacturer’s instructions. To evaluate the effects of proteinase treatment, we added a broad-spectrum serine proteinase, PK (Ambion, Austin, TX), to the plasma samples (final concentration: 0.5 or 1.0 mg/mL) before the precipitation. Plasma samples (0.6 mL) were mixed with PK by brief vortexing and incubated at 37 °C for 10 min. Samples with or without PK treatment were mixed with the precipitation buffer provided by the manufacturers and incubated at 4 °C for 30 min or 1 h following the manufacturers’ instructions. The precipitated pellets obtained by centrifugation (SBI, 1500× *g* for 30 min; LT and QG, 10,000× *g* for 5 min) were re-suspended in 200 μL PBS and used for total protein quantification or for Western blot analysis after dilution with radioimmunoprecipitation assay (RIPA) buffer. We evaluated the effect of plasma volume (0.6, 1.0, or 1.7 mL) on ELV enrichment by measuring the protein concentrations of isolated ELVs. Among the three tested kits, ELVs prepared with the QG kit and 0.5 mg/mL PK showed the optimal purity and yield (see results); hence, to isolate ELVs using acidification, we treated 0.6 mL plasma with 50 μL 1N HCl (1:12, v/v) after PK treatment, followed by the precipitation procedure. The workflow is illustrated in Figure 1.

### 2.3. Sodium Dodecyl Sulphate-Polyacrylamide Gel Electrophoresis (SDS-PAGE) and Western Blot Analysis

We characterized isolated ELVs in terms of the presence of proteins normally enriched in exosomes, including Alix, TSG-101, CD-63, or annexin-5, and the absence of GM130, calnexin, or apolipoprotein A-1 (apo A-1), proteins that are usually absent in exosomes. Quantification of total proteins in ELV preparations was performed using a bicinchoninic acid protein assay kit (Pierce, Rockford, IL) using ELVs in PBS, diluted five times (v/v) with RIPA buffer (50 mM Tris-HCl, 1% NP40, 150 mM NaCl, 1 mM EDTA, and 0.1% SDS). Twenty micrograms of protein in the ELV preparation were mixed with reducing sample buffer (50 mM Tris-HCl [pH 6.8], 10% glycerol, 2% SDS, 100 mM dithiothreitol, and 0.01% bromophenol blue) and separated on 10% SDS-PAGE gels. Proteins were then transferred to nitrocellulose membranes (#66485, Pall Corporation, Port Washington, NY) and incubated with blocking buffer containing 5% non-fat dried milk in 0.1% Tris-buffered saline-Tween 20 at RT for 1 h. We incubated membranes with primary antibodies (1:1000) against exosomal protein markers; that is, anti-TSG101 (M-19; Santa Cruz Biotechnology, #sc-6037), anti-CD63 (TS63; Abcam, #ab59479), anti-Annexin V (Abcam; #ab14196), and anti-Alix (3A9; Cell Signaling Technology, #2171), or non-exosomal proteins; that is, anti-GM130 (Clone 35/GM130; BD Transduction Laboratory, #610822), anti-calnexin (AF18; Santa Cruz Biotechnology, #sc-23954), and anti-apo A-1 (069-01; Santa Cruz Biotechnology, #sc-58230), at 4 °C overnight. Specific proteins were detected using an Enhanced Chemiluminescence Detection System (Pierce) or Immobilon Chemiluminescent HRP Substrate (Merck Millipore, Temecula, CA, USA). For quantitative analysis, we used a Chemidoc Touch Image system and Quantity One^®^ 4.6 image analysis software (Bio-Rad Laboratories, Inc., Hercules, CA, USA).

### 2.4. Morphological Characterization of ELVs by Electron Microscopy

ELVs were visualized using TEM as previously described, with slight modifications [32]. Briefly, ELV suspensions were diluted 1:50 with PBS. Two microliters of the fresh ELV solution were transferred onto Formvar-carbon-coated EM grids without fixation and allowed to dry at RT. The grids were then stained with 1% uranyl acetate for 1 min. Imaging was performed at an acceleration voltage of 75 KV using a charge-coupled device camera (5 × 5 k, Hamamatsu Photonics, Shizuoka, Japan) coupled to a H-7100 electron microscope (Hitachi High Technology, Tokyo, Japan).

### 2.5. Size Distribution Analysis of ELVs

Isolated ELVs were analysed using a Nanosight NS300 instrument with NTA3.2.16 software (Malvern, Worcestershire, UK) and dynamic light scattering (DLS; Zetasizer Nono ZS ZEN3600; Malvern) to measure the size distribution and number of particles. For the nanoparticle tracking analysis (NTA) by the Nanosight instrument, aliquots of the ELV suspensions were diluted in PBS (1:500) to achieve a particle concentration ranging from 10^6^ to 10^9^ particles/mL. Three runs were recorded for each sample. We compared the size distribution (mode) and number of particles among preparations. For the DLS, ELVs containing 100 μg of proteins were diluted in PBS (1 mL), and 3 × 10 measurement runs with standard settings (refractive index = 1.331, viscosity = 0.89, temperature = 25 °C) were performed, as previously described [32]. The intensity of the scattered light was measured at 175°. We compared the Z-average for size distribution from preparations measured in triplicate. Following typical DLS procedures, the peak intensity (% mass) can be detected over a wide range of size (~10,000 nm), which helps determine the purity of ELV preparations.

### 2.6. Extraction of RNAs and Amplification of Specific miRNAs

To evaluate the feasibility of isolating ELVs for miRNA research, we measured miRNAs in ELVs using qPCR. We isolated total RNA directly from the ELV pellets or plasma from six elderly persons using an RNA extraction kit (miRNeasy Serum/Plasma Advanced Kit, #217204; Qiagen) following the manufacturer’s instructions, with slight modifications. Briefly, 0.5 mL RNase-free water was added to the ELV pellets, then mixed by brief vortexing, and incubated for 5 min at RT. Plasma samples (0.5 mL) or lysed ELVs were mixed with RPL buffer (150 μL) for subsequent RNA elution. Samples mixed with the precipitation buffer (RPP buffer) were centrifuged at 12,000× *g* for 3 min at RT, and supernatants were transferred to new microfuge tubes and mixed with the same volume of isopropanol. The mixture was transferred to a miRNA spin column (RNeasy UCP MInElute column; Qiagen) and centrifuged at 8000× *g* for 15 s. After washing, the samples were eluted using 20 μL RNase-free water. cDNA was synthesized from 2 μL of extracted RNA using the miRCURY LNA RT Kit (Qiagen, #339340) following the manufacturer’s instructions. After reverse transcription, cDNA templates were diluted 1:40 and amplified with specific miRNAs. As there are no established ELV miRNAs that can be used as endogenous housekeeping controls, we measured the levels of miRNAs (i.e., miR-30c, miR-126, and miR-192) that have been reported to be abundant in ELVs [33]. We used primer sets for miR-30c-5p (#YP00204783), miR-126-3p (#YP00204227), and miR-192-5p (#YP00204099) from Qiagen, and carried out qPCR using a BioRad Prime PCR machine. The amplifying conditions using the miRCURY LNA SYBR GREEN PCR Kit (Qiagen, #339345) and primer sequences are summarized in Table 1. We simultaneously amplified the specific miRNAs extracted from total plasma or ELVs and compared the relative miRNA quantities using threshold cycle (Ct) values from plasma (pCt) and ELV (vCt).

### 2.7. Statistical Analysis

All data are presented as means ± standard errors. ELV protein quantities and expression levels of exosomal protein markers across the ELV purification methods were compared by the Mann–Whitney *U* test using GraphPad Prism software (version 6.0; GraphPad Software, San Diego, CA, USA). We considered *p* < 0.05 as significant.

## 3. Results

### 3.1. Molecular Characteristics of Isolated ELVs

**Figure 2:**
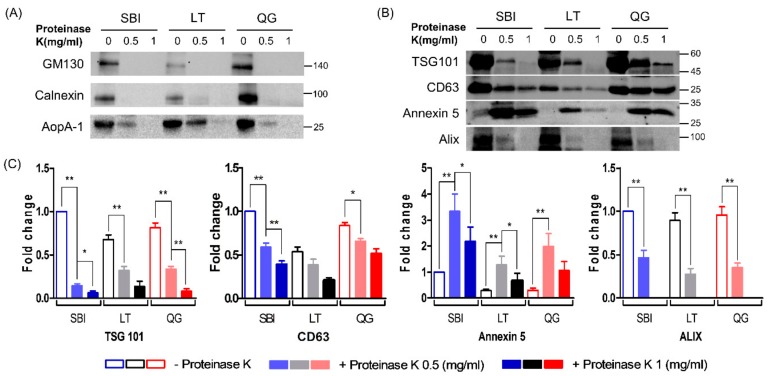
Western blot analyses of exosomal and non-exosomal proteins in ELV preparations using three kits with or without treatment of proteinase K. (**A**) Comparison of the expression levels of non-exosomal markers (GM130, calnexin, ApoA-1) in ELVs prepared by three kits. ELVs prepared without proteinase K showed the abundant expression of contaminated non-exosomal component, which was drastically decreased by treatment of proteinase K. A representative blot was shown (n = 6). (**B**) Expression of exosomal markers (TSG101, CD63, annexin 5, and Alix) in ELVs prepared by three kits with or without treatment of proteinase K. (**C**) Quantification of the expression levels of TSG101, CD63, annexin 5, and Alix in each ELV preparation showed that the levels of TSG101, CD63, and Alix were decreased by proteinase K treatment in a dose-dependent manner, while the level of annexin 5 was not (n = 6). * *p* < 0.05, ** *p* < 0.01 by Mann–Whitney U test. SBI, ExoQuick Exosome Isolation Kit for Serum and Plasma; LT, Invitrogen™ Total Exosome Isolation Kit; QG kit, miRCURY Exosome Serum/Plasma Kit.

We compared the expression levels of exosomal marker proteins (i.e., TSG101, CD63, and annexin 5) and non-exosomal proteins (i.e., GM130, calnexin, and apo A-1) in PK-treated and non-PK-treated ELV preparations. In ELVs prepared without PK, abundant expression of exosomal markers as well as non-exosomal markers were detected with all three kits. However, when samples were treated with PK, the levels of non-exosomal proteins were drastically reduced, although apo A-1 was scantly detected in ELV samples treated with 0.5 mg/mL of PK. The levels of exosomal marker proteins were also decreased by PK treatment, particularly at high concentrations of PK (1.0 mg/mL), except for annexin 5. With the SBI kit, the levels of exosomal markers per 20 μg ELV protein were higher than those of other kits when ELVs were prepared without PK. When samples were treated with 0.5 mg/mL PK, the levels of TSG101 in 20 μg ELV proteins were lower with the SBI than with the other kits, and the levels of Alix and CD63 were comparable with other kits, while the levels of annexin 5 were higher than those with the LT or QG preparation kit. The levels of annexin 5 in ELVs prepared with PK were significantly higher than those without PK. Treatment with high concentrations of PK (1 mg/mL) further reduced the level of exosomal markers in all kits. Alix was not detected in samples treated with 1 mg/mL of PK. Among the three tested kits, ELVs prepared with the QG kit and 0.5 mg/mL PK showed abundant expression of all measured exosomal markers (Figure 2).

### 3.2. Morphological Characterization of ELVs

**Figure 3:**
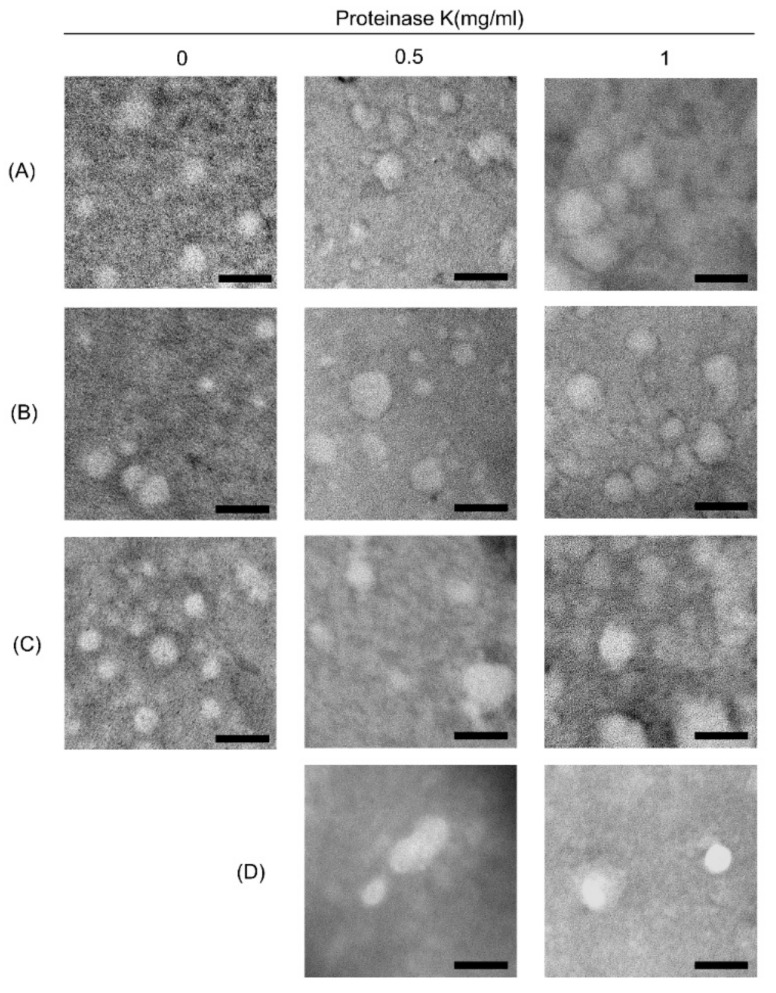
Electron microscopic image of exosomes following different isolation method. ELVs were purified using (**A**) SBI, (**B**) LT, (**C**,**D**) QIAGEN treated with different concentration of proteinase K (0, 0.5, 1mg/mL), and (**D**) additionally treated with HCl for acidification of samples to approximately pH 4.0. Diluted suspension containing ELVs was placed on formvar-carbon coated grid and negatively stained with uranyl acetate. Round-shaped vesicles with a heterogeneous size from 30 to 150 nm diameter were clearly visualized in all preparations. The horizontal bars indicate 100 nm.

In addition to the characterization of exosomal and non-exosomal protein expression in ELVs, we further characterized ELV preparations by morphological analysis using TEM. As shown in Figure 3, we clearly observed a round-shaped vesicular population with a heterogeneous size ranging from approximately 30 to 150 nm. When we added PK during preparation, the probable co-precipitates surrounding the untreated vesicles were mostly cleared. The morphology of ELVs in samples treated with HCl was not different from that of samples without acidification.

### 3.3. Comparison of Protein Wuantity According to ELV Isolation Methods

To compare the quantity of ELVs in the samples prepared by the different protocols, we measured the protein concentrations in ELVs prepared with or without PK, or HCl treatment.

As shown in Table 2, the mean protein concentrations in ELVs without PK treatment were significantly higher than those with treatment for all kits. The mean protein concentrations of ELV samples prepared using the QG kit with or without PK treatment were the highest, followed by the SBI and LT kit. The % coefficient of variation (CV) values for the protein concentrations of each preparation from the SBI, LT, or QG kit were 6.0%~8.9%, 15.9%~48.0%, and 5.1%~7.8%, respectively. In addition, acidification of samples followed by ELV precipitation using the QG kit and PK treatment increased the ELV protein concentration with low %CV values (< 3%) compared with the corresponding samples without acidification.

### 3.4. Effect of Acidification on the ELV Markers

**Figure 4:**
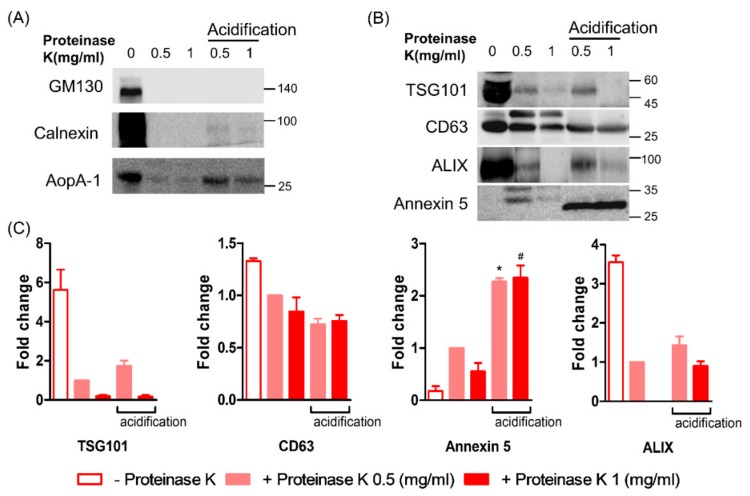
Effect of acidification on the level of exosomal and non-exosomal proteins in ELV preparation. (**A**) Levels of non-exosomal proteins (GM130, calnexin, and ApoA-1) were scanty by treatment of proteinase K, while the acidification slightly increased the expression of calnexin and ApoA-1, but not GM130. (**B,C**) Among the exosomal markers (TSG101, CD63, Alix, and annexin 5), acidification increased the level of TSG101, Alix, and annexin 5, as compared with non-acidified, but proteinase-treated ELVs preparation, while the level of CD63 was comparable. Representative bands were shown (n = 6). * *p* < 0.05 vs. ELV with proteinase K 0.5 mg/mL without acidification; # <0.05 vs. ELV with proteinase 1.0 mg/mL without acidification, by Mann–Whitney *U* test.

In a previous study, acid treatment of cell culture media during ELV preparation significantly increased the abundance of exosomal markers in the preparations [34]. In concordance with this, we also observed an increase in the abundance of exosomal marker proteins by acidification of PK-treated samples (Figure 4). Although the expression levels of CD63 in acid-treated samples were comparable to non-acidified, but PK-treated samples, the levels of other exosomal markers (i.e., Annexin 5, TSG101, and Alix) were increased by HCl treatment, as compared with those in non-acidified, but PK-treated samples. In addition, non-exosomal markers were slightly increased by acidification, although GM130 was still not detected.

### 3.5. ELV Protein Concentrations According to Plasma Volume

As the QG kit showed the highest protein concentrations with the lowest %CV, and all tested exosomal marker proteins were detected, we tested the effect of plasma volume on ELV protein concentrations using the QG kit. For clinical application, the required plasma volume needs to be optimized. We compared the ELV protein concentrations in PK-treated preparations from 0.6, 1.0, and 1.7 mL plasma. Regardless of the starting volume, the ELV pellets were resuspended in 200 μL of PBS. The mean concentrations of ELV protein (n = 4) normalized by volume of plasma (per mL) were 12.6, 10.7, and 8.8 mg/mL, and the %CVs of the protein concentrations were 9.2%, 7.6%, and 7.8% for 0.6, 1.0, and 1.7 mL of plasma, respectively.

### 3.6. Size Distribution of ELV Preparations

**Figure 5:**
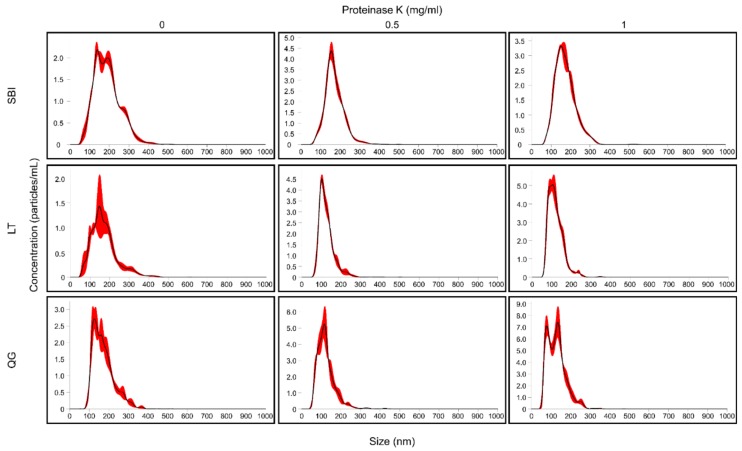
Size distribution and quantification analysis of ELV preparation by nanoparticle tracking analysis (NTA). The number of particles (×10^7^) per mL and size distribution for each preparation (1:500 dilution) is shown. The calculated size distribution of particles is depicted as a mean (black line) with standard error (red shaded area).

As shown in Figure 5 and Table 3, when ELVs were prepared without PK treatment, we observed several subpopulations of differently sized vesicles with multiple concentration peaks in the NTA (mean sizes: 188.8, 171.8, and 172.5 nm for the SBI, LT, and QG kits, respectively). However, the treatment of samples with 0.5 mg/mL PK slightly reduced the sizes of vesicles with a homogeneous concentration peak (mean size: 170.8, 126.2, and 125.4 nm for the SBI, LT, and QG kits, respectively). In ELVs prepared with the higher PK concentration (1 mg/mL), we observed a mean size and number of vesicles similar to those of ELVs prepared with 0.5 mg/mL PK, except for the QG kit (two modes of the concentration peaks). A positive effect of the PK treatment on the homogeneity of vesicle seizes was also observed in the DLS analysis. In all kits, PK treatment reduced the heterogeneity of the size distribution (Figure S1).

### 3.7. Relative Quantification of miRNAs in Total Plasma or Isolated ELVs

**Figure 6:**
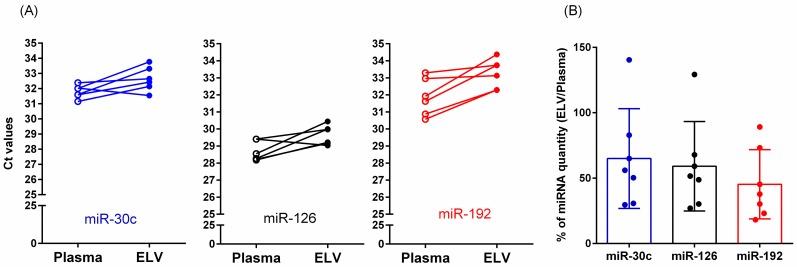
Quantification of selected miRNAs in plasma or ELV using quantitative PCR. (**A**) Ct vaules of each miRNA extracted from plasma or ELV (n = 6) ranged from 28 to 34 cycles. Ct values for ELV miRNA amplification were higher than those for miRNA amplification using total plasma. (**B**) Relative quantities miRNAs (ELV/Plasma) are shown. Vertical bars and lines indicate mean and standard deviation, respectively.

On the basis of our results of ELV preparations with PK treatment (high yield, purity, and consistency), we tested the feasibility of our method for clinical research using vesicular miRNAs. Because we treated plasma with PK, it is likely that ectodomains of membranous proteins spanning across ELV membranes, as well as possibly contaminating proteins, might have been degraded. Therefore, our method was considered to be more suitable to detect intra-vesicular proteins or nucleic acids. To test this, we evaluated the relative levels of miRNAs (Ct values) enriched in ELVs (i.e., miR-30c, miR-126, and miR-192) using the PK-treated QG kit method with those found in total plasma measured with qPCR. We measured all tested miRNAs successfully in the ELV preparations from the plasma of cognitively normal elderly subjects and patients with AD (Figure 6). Compared with the miRNA quantities in total plasma (Ct values of plasma miRNAs), the mean quantities of miRNAs in prepared ELVs (Ct values of ELV miRNAs) were low (2^(vCt–pCt)^ × 100 = 45–65%). The within-run variability of duplicate measures of miRNAs in plasma and ELVs was very low (mean %CVs < 1%). Interindividual variations of Ct values for total plasma (1.4%–3.4%) and ELV (1.9%–2.6%) miRNA amplifications were comparable.

## 4. Discussion

The aim of this study was to develop an optimized method for isolating ELVs with high yield and purity from human plasma for ELV-associated miRNA research. We evaluated multiple characteristics of isolated ELVs and found that treatment of plasma with PK improved the purity of ELVs prepared using commercially available PP-based ELV isolation kits. Furthermore, our method can successfully quantify selected miRNAs in ELVs, indicating that the modified method may reduce bias and variability by unintended contamination with non-vesicular proteins and miRNAs.

Circulating extracellular miRNAs have been identified in all biofluids, and there is increasing evidence that subsets of miRNAs are associated with EVs, including exosomes [35,36,37]. miRNAs in ELVs of certain biofluids, particularly plasma or serum, have been suggested as disease biomarkers [18,19,20,38]. However, to validate miRNA biomarkers, it is essential to isolate ELVs with high yield and purity, but low variability from biofluids, and to control pre-analytical variables. Recent studies have suggested that the yield of ELV-associated RNA from 1 mL biofluid is less than 5 ng [39]; hence, ELV isolation methods with high purity, but low yield (e.g., filtration-based methods or DG-UC) may limit their clinical application. Plasma is a complex matrix that contains a variety of proteins, lipids, and circulating nucleic acids. In particular, a variety of proteins can be co-precipitated during ELV isolation using PP methods, which can interfere with downstream analyses [40]. Circulating miRNAs are protected by non-vesicular protein complexes, in addition to vesicular packaging [4,41]. Furthermore, during blood collection and preparation, certain cellular components can be contaminated by microhaemolysis. Considering the purity of ELVs, we observed that ELVs isolated by commercially available exosome isolation kits without PK treatment showed an abundance of apo A-1, calnexin, and GM130, which is in agreement with a previous study [22]. This result indicates that ELVs prepared using commercial PP kits without additional measures are contaminated with non-vesicular components (i.e., apo A-1, which indicates contamination with extracellular miRNA-carrying lipoproteins such as HDL; and calnexin and GM130, which indicate contamination with intracellular organelles); hence, subsequent miRNA analyses may produce biased results. Fortunately, we found that treatment of plasma with PK before the precipitation procedure drastically reduced the amount of apo A-1, calnexin, and GM130 contamination with all tested kits, indicating that our method significantly removed unintended contamination with non-vesicular proteins. In fact, the polyethylene glycol PP reagent is not specific for precipitating EVs, but sterically excludes the macromolecular complexes from water molecules, allowing them to be precipitated easily by low-speed centrifugation [42]. Therefore, the proteolysis of extravesicular proteins by PK treatment may prevent co-precipitation of proteins.

Considering the yield of ELVs, treatment of plasma with PK significantly reduced the level of exosomal markers and protein concentrations compared with PK-free preparations. However, the removal of contaminating non-vesicular proteins by PK treatment may compromise samples by increasing homogeneity, which causes the reduction of exosomal markers. It can be speculated that the reduced levels of Alix, TSG101, and CD63 in PK-treated samples may be caused by a possible partial disruption of the exosomal membrane integrity by cleavage of the ectodomains of vesicular transmembrane proteins. However, we could not conclude that our method dramatically reduced the recovery of ELVs [43,44]. We clearly observed intact ELVs in the TEM analysis, and even increased expression of another exosomal marker, annexin 5, in ELVs from PK-treated samples; this was not the case for non-treated samples. The results of TEM and immunoblot analysis may be contradictory to the hypothesis of partial disruption of ELVs by PK treatment. In addition, PK treatment improved the homogeneity of vesicles from a broad range of sizes and multiple subpopulations to a neat preparation of vesicles with a homogenous size distribution. Therefore, treatment of PK may change the precipitation efficiency of vesicles by kits according to the molecular or physical characteristics of subpopulation of ELVs [45,46], although our hypothesis should be further investigated. Interestingly, we observed a higher peak concentration of ELV-sized vesicles in PK-treated preparations than in untreated samples. The improvement of resolution by PK treatment in NTA can be explained by the dissociation of aggregated particles or removal of co-aggregated non-vesicular particles. However, considering the yield of ELVs, the NTA results do not exactly agree with the Western blot results, indicating that the effects of PK on ELV stability and yield need to be investigated further [2]. The acidification of samples with HCl (approximately pH 4.0) using the QG kit, which showed the lowest variability (<8%), but the highest protein concentrations (1.5–13.4 times that of other kits) of ELV isolates, significantly preserved the expression levels of exosomal markers. Ban et al. reported that a low pH increased the yield of exosome isolates [34]; however, they did not provide data on possible contamination of non-vesicular protein complexes. Here, we observed that acidification increased the recovery of ELV markers in PK-treated samples without significant loss of sample purity, although the acidification showed slightly increased levels of calnexin and apo A-1 compared with non-acidified samples. Therefore, our ELV isolation method may be suitable to investigate circulating ELV-associated miRNAs with minimal non-vesicular impurities in plasma. Furthermore, our ELV isolation method showed acceptable variability (< 10%) when measuring ELV protein quantities in isolates.

To test the feasibility of ELV miRNA analyses, we measured the expression of selected miRNAs (i.e., miR-30c, miR-126, and miR-192) that are abundant in ELVs and compared them with the levels of total circulating miRNAs [33]. We found that these miRNAs were highly expressed in ELVs isolated from a small plasma volume (0.6 mL) with consistent levels across disease statuses (cognitively normal elderly persons and AD patients). However, the sample size was very limited. Furthermore, the quantification of miRNAs in ELVs prepared by our method showed excellent reproducibility, which may warrant its clinical applicability. The lower quantity of ELV miRNAs compared with the miRNA quantities in total plasma may indicate that treatment with PK during ELV preparation may eliminate non-vesicular miRNAs (e.g., HDL-associated miRNAs). The difference of relative quantities of miRNA in ELVs compared with total plasma may also be explained by the different intrinsic degrees of encapsulation according to the specific miRNAs. We did not use internal standards for normalization of miRNA amplification by qPCR because there are no well-accepted control transcripts available for data normalization. Further studies will thus be needed once these control transcripts have been developed. Collectively, ELV preparations using a commercially available PP kit combined with PK treatment may be a reliable method for ELV miRNA research in a clinical setting.

Our study has several limitations. First, we did not measure the effects of PK treatment on the purity and yield of ELVs or the quantification of miRNAs in a large number of samples with various clinical characteristics. Given that there is no valid plasma miRNA biomarkers in ELVs for AD diagnosis, we tested the clinical feasibility of our method using samples from elderly subjects. The number of clinical samples was very low (i.e., only six plasma samples from elderly healthy or Alzheimer’s disease subjects), hence our method could not warrant applicability to clinical studies for various diseases. Therefore, our method should be further validated for consistency. Nevertheless, our results showed a reduction of possible contamination with non-vesicular proteins and miRNAs by a simple modification of protocols of widely used commercial kits. Second, the number of tested miRNAs was limited. In this study, we aimed at testing the feasibility of our method for miRNA research; therefore, the clinical applicability should be further evaluated in clinical samples with various diseases and a variety of miRNAs. Third, although we observed non-exosomal markers, GM130 and calnexin could not be detected by Western blot analysis in our PK-treated ELV preparation; our results do not warrant the complete elimination of possible impurities from non-vesicular miRNAs, such as apo A-1. It is plausible that different ELV isolation methods can be applied for different downstream analyses; therefore, our method may be applicable for miRNA studies for samples with tolerable impurities. In addition, our method for ELV isolation and miRNA quantification can be further improved by combining it with other ELV enrichment methods.

## 5. Conclusions

In summary, we found that treatment of plasma with PK significantly improved the purity of ELVs isolated using commercially available kits. In addition, acidification of plasma samples followed by PK treatment increased the levels of ELV markers. Our enrichment method of ELVs from small volumes of human plasma appears to be an easy, consistent, and clinically feasible tool for ELV miRNA research. Although further studies in larger clinical sample sets with various disease states are necessary, our method appears suitable for quantifying ELV-associated miRNAs using qPCR.

## Figures and Tables

**Table 1:** Quantitative PCR condition for amplification of the specific miRNAs.

miRNA	Target Sequences	Number of Cycles	PCR Condition
miR-30c-5p	5′-UGUAAACAUCCUACACUCUCAGC-3′	40	PCR initial heat activation; 95 °C, 2 min. Denaturation; 95 °C, 10 sec. Combined anneling/extention; 56 °C, 1 min.
miR-126-3p	5′-UCGUACCGUGAGUAAUAAUGCG-3′	40	
miR-192-5p	5′-CUGACCUAUGAAUUGACAGCC-3′	40	

**Table 2:** Comparison of protein concentration and its variability according to the exosome-like extracellular vesicle (ELV) preparing methods. QG, miRCURY Exosome Serum/Plasma Kit; SBI, ExoQuick Exosome Isolation Kit for Serum and Plasma; LT, Invitrogen™ Total Exosome Isolation Kit.

Kit	SBI (n = 6)			LT (n = 6)			QG (n = 6)			QG (n = 4)	
Acidification	-	-	-	-	-	-	-	-	-	+	+
Proteinase K (mg/mL)	0	0.5	1	0	0.5	1	0	0.5	1	0.5	1
Protein concentration (mean, mg/mL)	9.7	8.8	7.8	5.8	1.8	0.9	16.1	13.0	12.1	21.7	20.3
%CV	8.9	6.0	8.3	15.9	26.5	48.0	5.1	7.4	7.8	2.4	2.6

**Table 3:** Size distribution of ELVs analyzed by nanoparticle tracking analysis (NTA) and dynamic light scattering (DLS) analysis.

Kit	Proteinase K (mg/mL)	DLS		NTA
		Z-Average (d.nm)	Polydispersity Index (PdI)	Size (Mode ± SD, nm)
SBI	0	106.87	0.56	160.0 ± 17.0
	0.5	130.10	0.29	151.2 ± 4.9
	1	129.97	0.27	158.1 ± 6.0
LT	0	399.23	0.56	149.2 ± 17.5
	0.5	95.70	0.22	101.6 ± 1.1
	1	95.38	0.19	101.2 ± 7.1
QG	0	76.98	0.54	126.4 ± 5.6
	0.5	73.57	0.28	110.5 ± 6.7
	1	90.22	0.23	110.8 ± 16.9

## Supplementary Materials

The following are available online at https://www.mdpi.com/2077-0383/8/11/1995/s1, Figure S1: Size distribution analysis using the Dynamic Light Scattering (DLS); Table S1: Demographic information and clinical diagnosis of volunteers who provided plasma samples for miRNA analyses.
