# Supplementary material for: Enrichment of Exosome-Like Extracellular Vesicles from Plasma Suitable for Clinical Vesicular miRNA Biomarker Research
Source: J Clin Med. 2019 Nov 15;8(11):1995. doi: 10.3390/jcm8111995 (PMC6912341; doi:10.3390/jcm8111995)
Supplement: Supplementary file 1 [file jcm-08-01995-s001.pdf]

Suplementary Materials

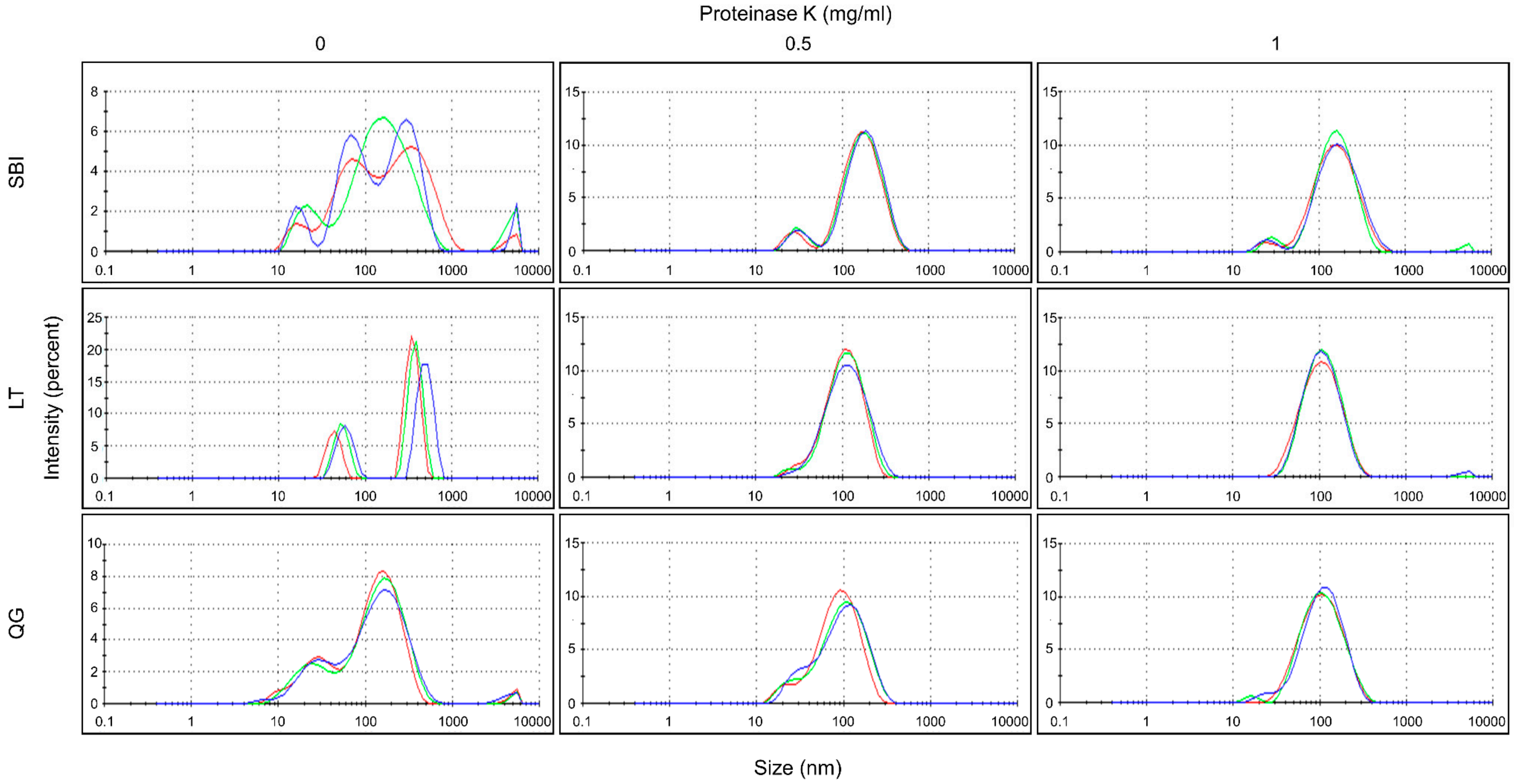

Supplementary Figure S1. Size distribution analysis using the Dynamic Light Scattering (DLS). Using the QG kit, size of isolated ELVs with or without proteinase K (PK) treatment was measured by DLS analysis. Without PK treatment, multiple peaks of intensity were observed, however, PK treatment prior to precipitation increased the homogeneity of the size of vesicles in all 3 kits.

Supplementary Table S1. Demographic information and clinical diagnosis of volunteers who provided plasma samples for miRNA analyses.

Sample	Age	Gender	Level of Education (years)	Diagnosis	Amyloid PET
NL1	58	F	12	Normal cognitive function	Negative
NL2	68	F	12	Normal cognitive function	Negative
NL3	67	F	0.5	Normal cognitive function	Negative
AD1	85	F	6	Alzheimer's dementia	Positive
AD2	76	F	9	Alzheimer's dementia	Positive
AD3	85	F	0.5	Alzheimer's dementia	Positive
